# Supplementary material for: Phospholipid translocation captured in a bifunctional membrane protein MprF
Source: Nat Commun. 2021 May 18;12:2927. doi: 10.1038/s41467-021-23248-z (PMC8131360; doi:10.1038/s41467-021-23248-z)
Supplement: Supplementary file 1 — Supplementary Information [file 41467_2021_23248_MOESM1_ESM.pdf]

**Phospholipid Translocation Captured in a Bifunctional Membrane Protein MprF**

Danfeng Song[1,2,†], Haizhan Jiao[1,2,†] and Zhenfeng Liu[1,2,*]

[1]National Laboratory of Biomacromolecules, CAS Center for Excellence in Biomacromolecules, Institute of Biophysics, Chinese Academy of Sciences, 15 Datun Road, Chaoyang District, Beijing 100101, P. R. China.

[2]College of Life Sciences, University of Chinese Academy of Sciences, No. 19(A) Yuquan Road, Shijingshan District, Beijing 100049, P. R. China.

[†]These authors contributed equally to this work: Danfeng Song, Haizhan Jiao.

[*]Correspondence and requests for materials should be addressed to Zhenfeng Liu (liuzf@ibp.ac.cn).

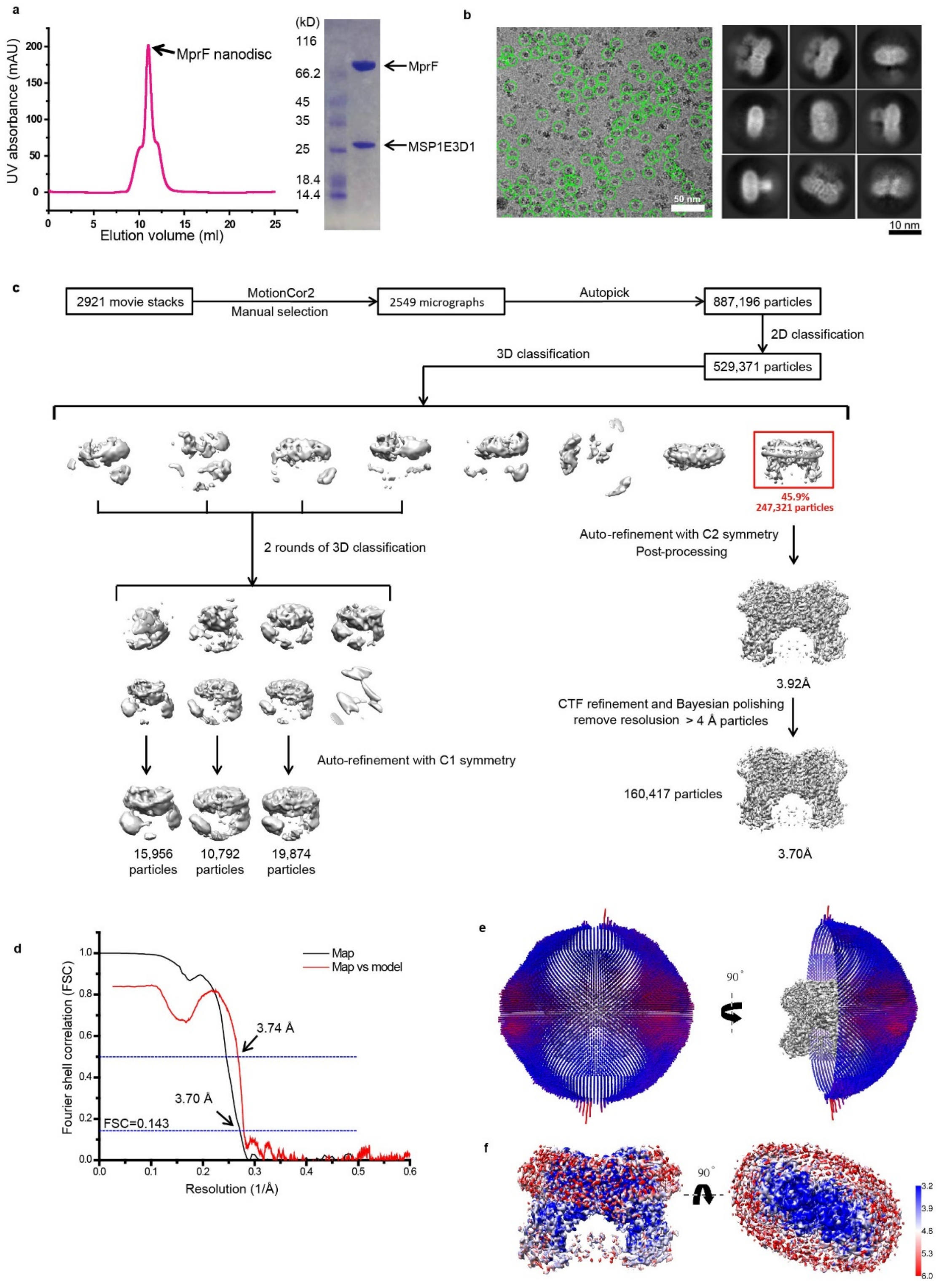


**Supplementary Figure 1. Sample preparation, cryo-EM data processing of *Rt*MprF(DDM) reconstituted in nanodiscs. a,** Purification of *Rt*MprF(DDM)-nanodisc samples through gel filtration and further analysis of the peak fraction through SDS-PAGE. The experiment was repeated independently four times with similar results.

**b**, A representative cryo-EM image of the sample (scale bar 50 nm) and 2D class images of *Rt*MprF in nanodiscs (scale bar 10 nm). A total of 2921 independent images of similar quality were collected and used for further data processing. The 2D class images shown are nine different class average results from a total of 529,371 particle images. **c**, A flow chart of data processing, 3D classification and refinement process. **d**, The Fourier Shell Correlation (FSC) curves. Black, the gold standard FSC curve between two independent half maps; red, the FSC curve between the map and structural model. **e**, Angular distribution of the major class of particles included in the final 3D reconstruction of the map. **f**, Estimation of the local resolution of the final cryo-EM map. The unit for the numbers labeled nearby the gradient color bar is Å.

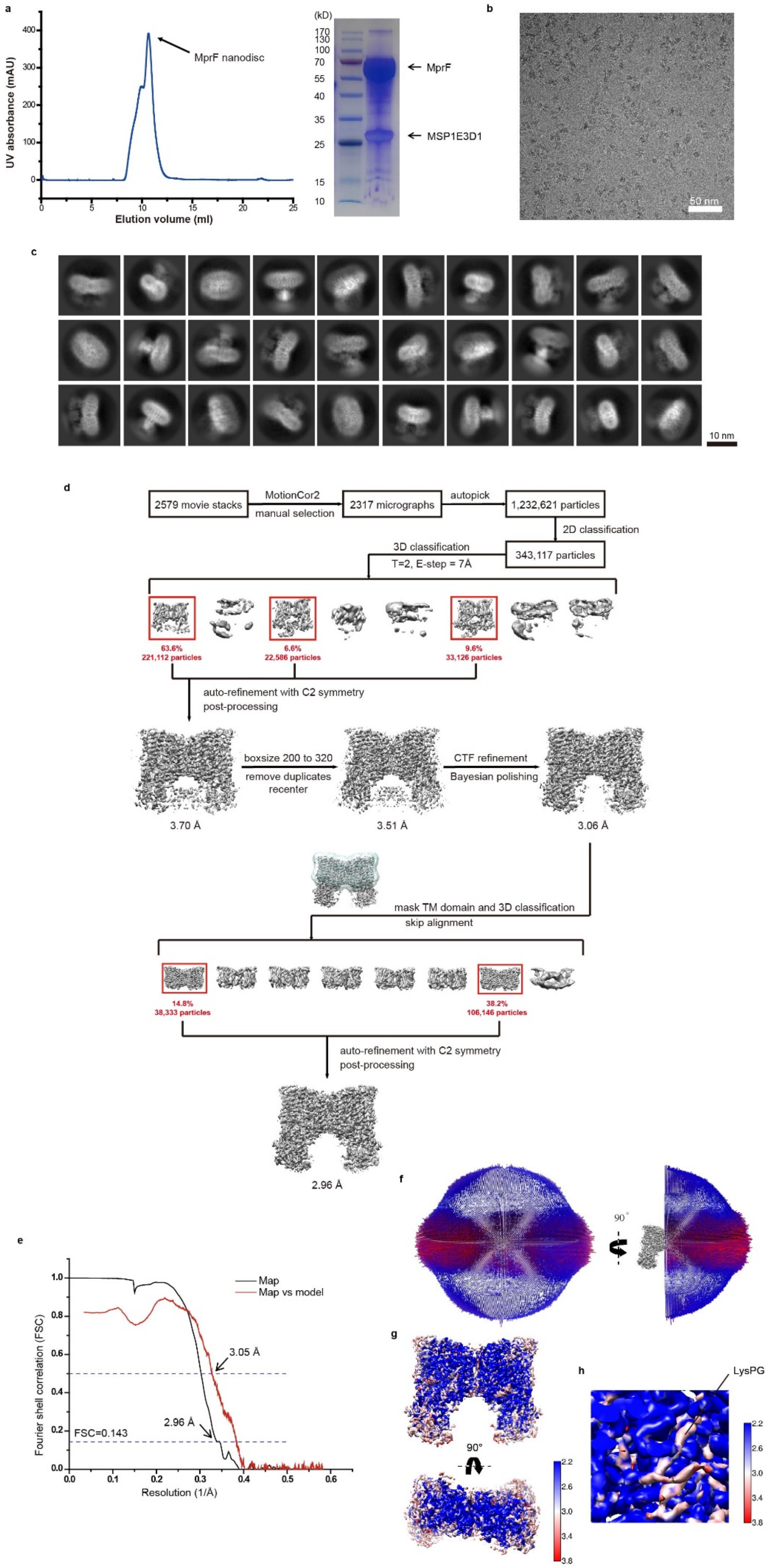
a
UV absorbance (mAU)
Elution volume (ml)
MprF nanodisc
(kD)
MprF
MSP1E3D1
b
50 nm
c
10 nm
d
2579 movie stacks
MotionCor2
manual selection
2317 micrographs
autopick
1,232,621 particles
2D classification
343,117 particles
3D classification
T=2, E-step = 7Å
63.6%
221,112 particles
6.6%
22,586 particles
9.6%
33,126 particles
auto-refinement with C2 symmetry
post-processing
boxsize 200 to 320
remove duplicates
recenter
CTF refinement
Bayesian polishing
3.70 Å
3.51 Å
3.06 Å
mask TM domain and 3D classification
skip alignment
14.8%
38,333 particles
38.2%
106,146 particles
auto-refinement with C2 symmetry
post-processing
2.96 Å
e
Fourier shell correlation (FSC)
Resolution (1/Å)
Map
Map vs model
3.05 Å
2.96 Å
FSC=0.143
f
90°
g
90°
h
LysPG

**Supplementary Figure 2. Sample preparation, cryo-EM data processing of *Rt*MprF(GDN) reconstituted in nanodiscs. a,** Purification of *Rt*MprF(GDN)-nanodisc samples through gel filtration and further analysis of the peak fraction through SDS-PAGE. The experiment was repeated independently four times with similar results. **b**, A representative cryo-EM image of the sample. Scale bar 50 nm. A total of 2579 independent images of similar quality were collected and used for further data processing. **c**, 2D class images of *Rt*MprF in nanodiscs. Scale bar 10 nm. The images shown are 30 different class average results from a total of 343,117 particle images. **d**, A flow chart of data processing, 3D classification and refinement process. **e**, The Fourier Shell Correlation (FSC) curves. Black, the gold standard FSC curve between two independent half maps; red, the FSC curve between the map and structural model. **f**, Angular distribution of the major class of particles included in the final 3D reconstruction of the map. **g**, Estimation of the local resolution of the final cryo-EM map. **h.** The local resolution map around the substrate-binding site in Cavity C with a LysPG molecule bound.

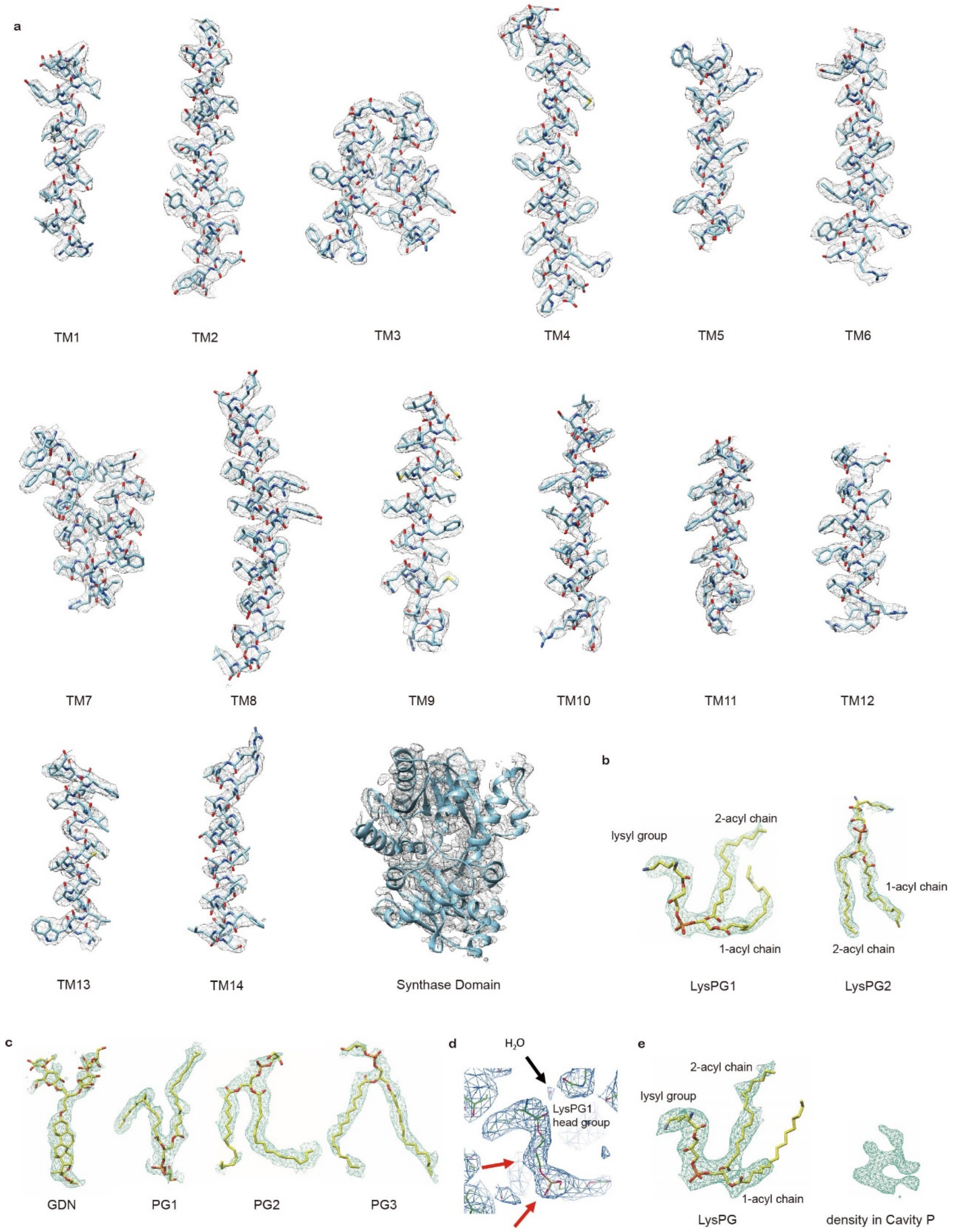


**Supplementary Figure 3. Representative cryo-EM densities of different regions of *Rt*MprF and the associated lipid molecules. a,** Cryo-EM densities for the 14 transmembrane helices and the C-terminal synthase domain (CTD) in *Rt*MprF(GDN)-nanodisc structure. All of the density features shown are from the postprocessed map with a default sharpening B-factor of -62 Å$^2$. **b,** Cryo-EM densities for the two LysPG

molecules in Cavity C and Cavity P of *Rt*MprF(GDN)-nanodisc structure. **c**, Cryo-EM densities for GDN and two phosphatidylglycerol molecules (PG1 and PG3) located at the dimerization interface of *Rt*MprF(GDN)-nanodisc and PG2 at the dimerization interface of *Rt*MprF(DDM)-nanodisc. The *Rt*MprF(DDM)-nanodisc structure contains a PG1 density highly similar to the corresponding one in *Rt*MprF(GDN)-nanodisc. The binding sites of GDN and PG3 from *Rt*MprF(GDN)-nanodisc partially overlaps with PG2 from *Rt*MprF(DDM)-nanodisc. **d,** A zoom-in view of the local region around the head group of LysPG1 molecule in *Rt*MprF(GDN)-nanodisc. The red arrows indicate the pointy features characteristic of the polar head group, distinguishing itself from the fatty acyl chains. For visualization of the local details, the cryo-EM map is contoured at 1.7 rmsd. **e,** Cryo-EM densities for the molecules in Cavity C and Cavity P of *Rt*MprF(DDM)-nanodisc. In comparsion, the density in Cavity P of *Rt*MprF(DDM)-nanodisc is much weaker than the one in Cavity P of *Rt*MprF(GDN)-nanodisc (b).

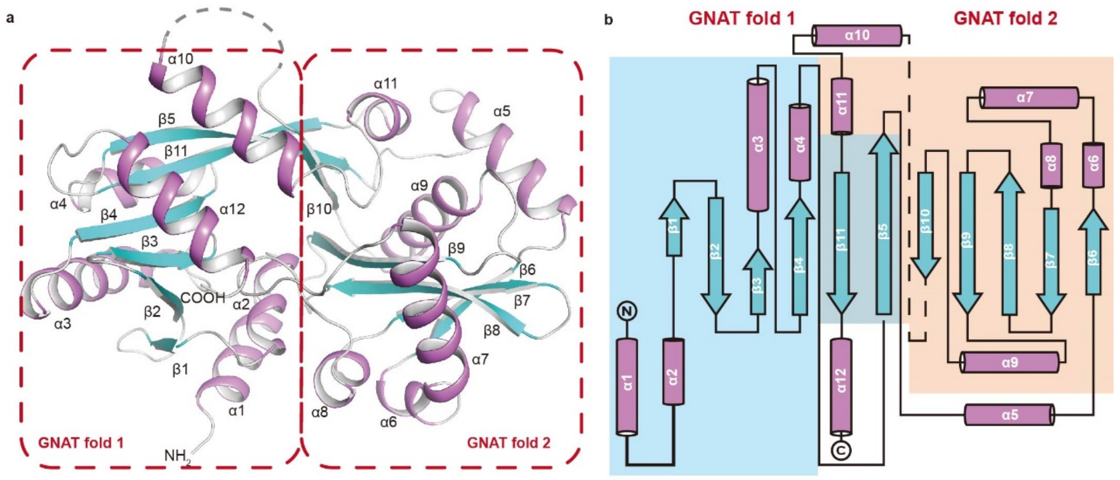


c

| | *Rt*MprF-SD Native | *Rt*MprF-CTD SeMet Derivative |
|---|---|---|
| **Data Collection** | | |
| Space group | $C222_1$ | $C222_1$ |
| Cell dimensions | | |
| *a, b, c* (Å) | 83.588, 95.793, 211.242 | 83.720, 95.937, 211.972 |
| *α, β, γ* (°) | 90, 90, 90 | 90, 90, 90 |
| Resolution (Å) | 50.00-2.00 (2.07-2.00) | 50.00-2.47 (2.56-2.47) |
| $R_{merge}$ | 0.047 (0.195) | 0.048 (0.136) |
| $I/\sigma(I)$ | 35.5 (8.3) | 60.6 (21.3) |
| Completeness (%) | 99.9 (99.7) | 99.9 (100) |
| Redundancy | 6.2 (5.8) | 17.0 (16.0) |
| **Refinement** | | |
| Resolution(Å) | 35.207-1.998 (2.070-1.998) | |
| No. reflections | 57555 (5564) | |
| $R_{work}/R_{free}$ | 16.83/19.84 (19.74/25.37) | |
| No. of atoms | | |
| All | 5683 | |
| Protein | 4956 | |
| Water | 727 | |
| B-factors(Å$^2$) | | |
| All | 27.4 | |
| Protein | 26.2 | |
| Water | 36.1 | |
| R.m.s deviations | | |
| Bond lengths (Å) | 0.0075 | |
| Bond angles (°) | 0.85 | |

**Supplementary Figure 4. Crystal structure of the synthase domain of *Rt*MprF. a,** The cartoon model of the synthase domain. The α-helices and β-strands are colored violet and cyan, respectively. The red dash boxes define the regions belonging to GNAT folds 1 and 2. **b,** Topology of the synthase domain. The α-helices and β-strands are indicated as cylinders (violet) and arrows (cyan), respectively. GNAT folds 1 and 2 are highlighted in blue or orange background. **c**, Data collection and refinement statistics for crystal structure of *Rt*MprF-SD. One crystal each was used for collecting the native and Se-Met derivative data. Values in parentheses are for highest-resolution shell.

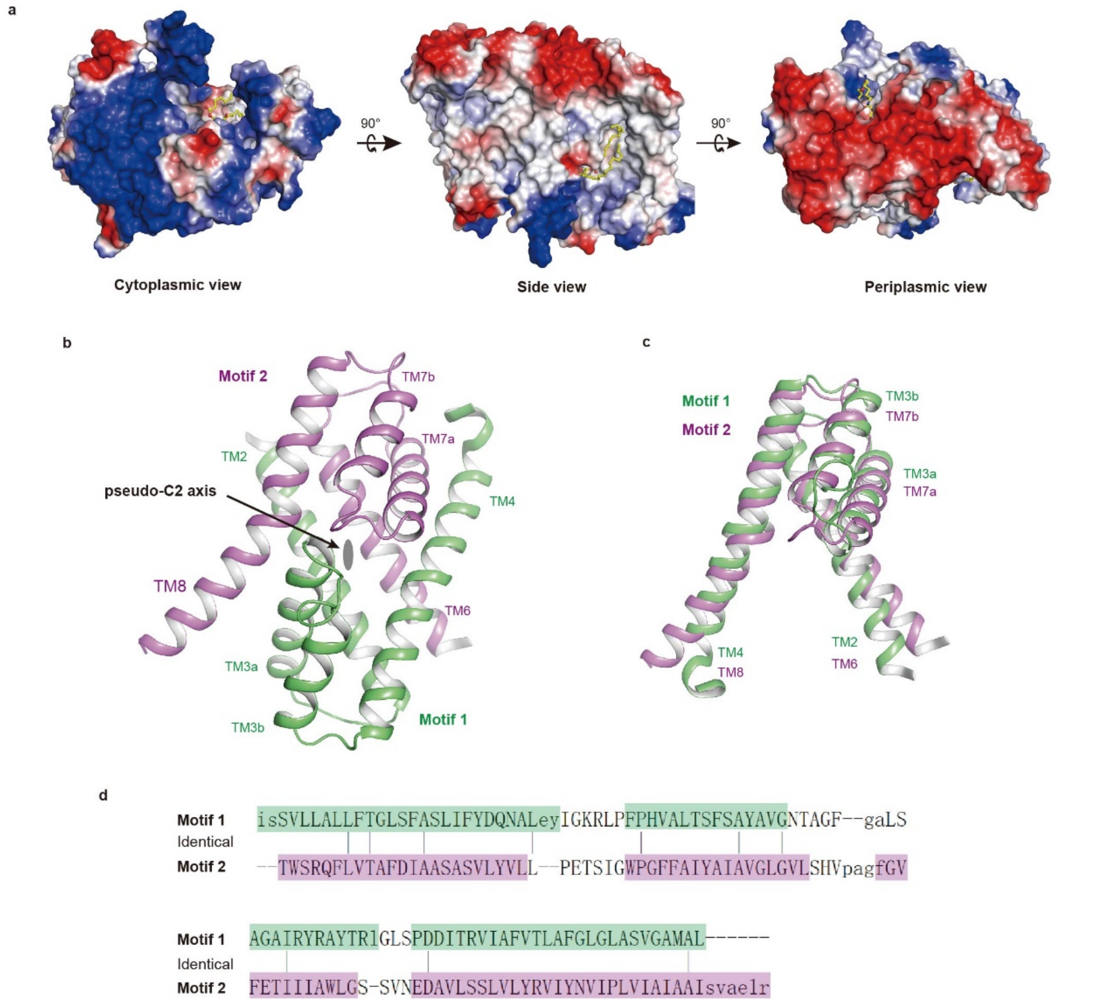


**Supplementary Figure 5. Surface model of the flippase domain and presence of two inverted repeat motifs. a,** Electrostatic potential surface of the flippase domain of *Rt*MprF**.** Blue, positive potential; white, neutral; red, negative potential. Left**,** cytoplasmic view; middle, side view; right, periplasmic view. The LysPG molecule is shown as stick model in yellow. **b**, Motifs 1 and 2 related by a pseudo-*C*2 axis. Motifs 1 and 2 are colored in green and violet respectively. **c**, Superposition of Motif 1 with Motif 2. **d,** Structure-based sequence alignment of the two motifs based on the output from the Dali server.

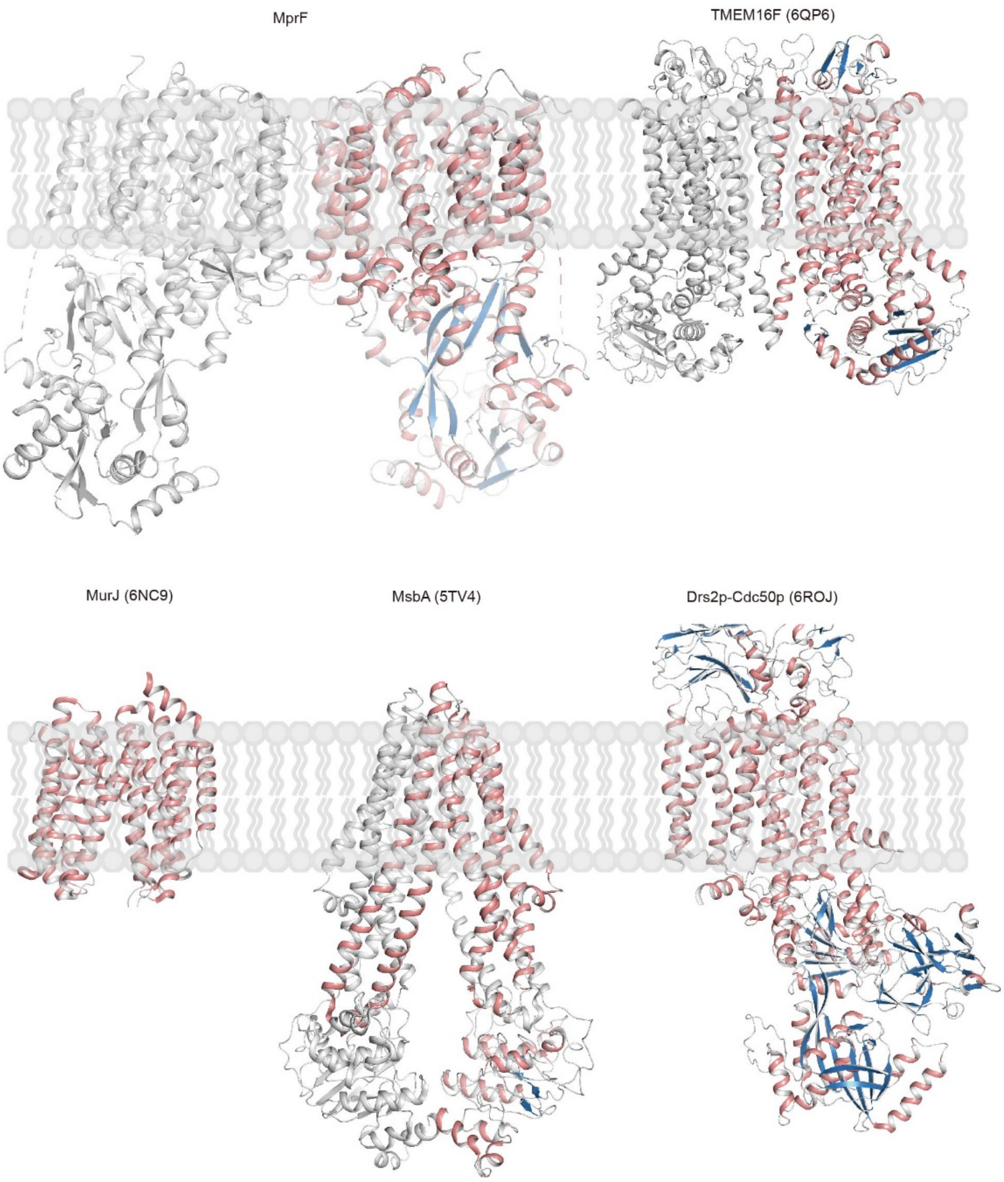


**Supplementary Figure 6. Comparison of *Rt*MprF structure with those of other lipid transporters.** The structures of *Rt*MprF, TMEM16F [PDB: 6QP6 (https://www.rcsb.org/structure/6QP6)], MurJ [PDB: 6NC9 (https://www.rcsb.org/structure/6NC9)], MsbA [PDB: 5TV4 (https://www.rcsb.org/structure/5TV4)] and Drs2p-Cdc50p [PDB: 6ROJ (https://www.rcsb.org/structure/6ROJ)] are shown as cartoon models. The $\alpha$-helices and $\beta$-strands are colored in red and blue, respectively. TMEM16F is a calcium-activated ion channel and lipid scramblase catalyzing translocation of

phosphatidylserine. MurJ is a secondary active transporter and belongs to Multidrug/Oligosaccharidyllipid/Polysaccharide (MOP) exporter superfamily, it might translocate Lipid II (the lipid-linked peptidoglycan precursor) by coupling the movement of an ion down its electrochemical gradient. MsbA is an ABC transporter and translocate Lipid A (the lipid component of bacterial lipopolysaccharides) from the inner to the outer leaflet of the inner membrane in gram negative bacteria. Drs2p-Cdc50p is a P4-type ATPase which is responsible for transporting phospholipids (such as phosphatidylserine, etc) in eukaryotic cells. Both MsbA and Drs2p-Cdc50p are primary active transporters and use ATP hydrolyzing as energy source.

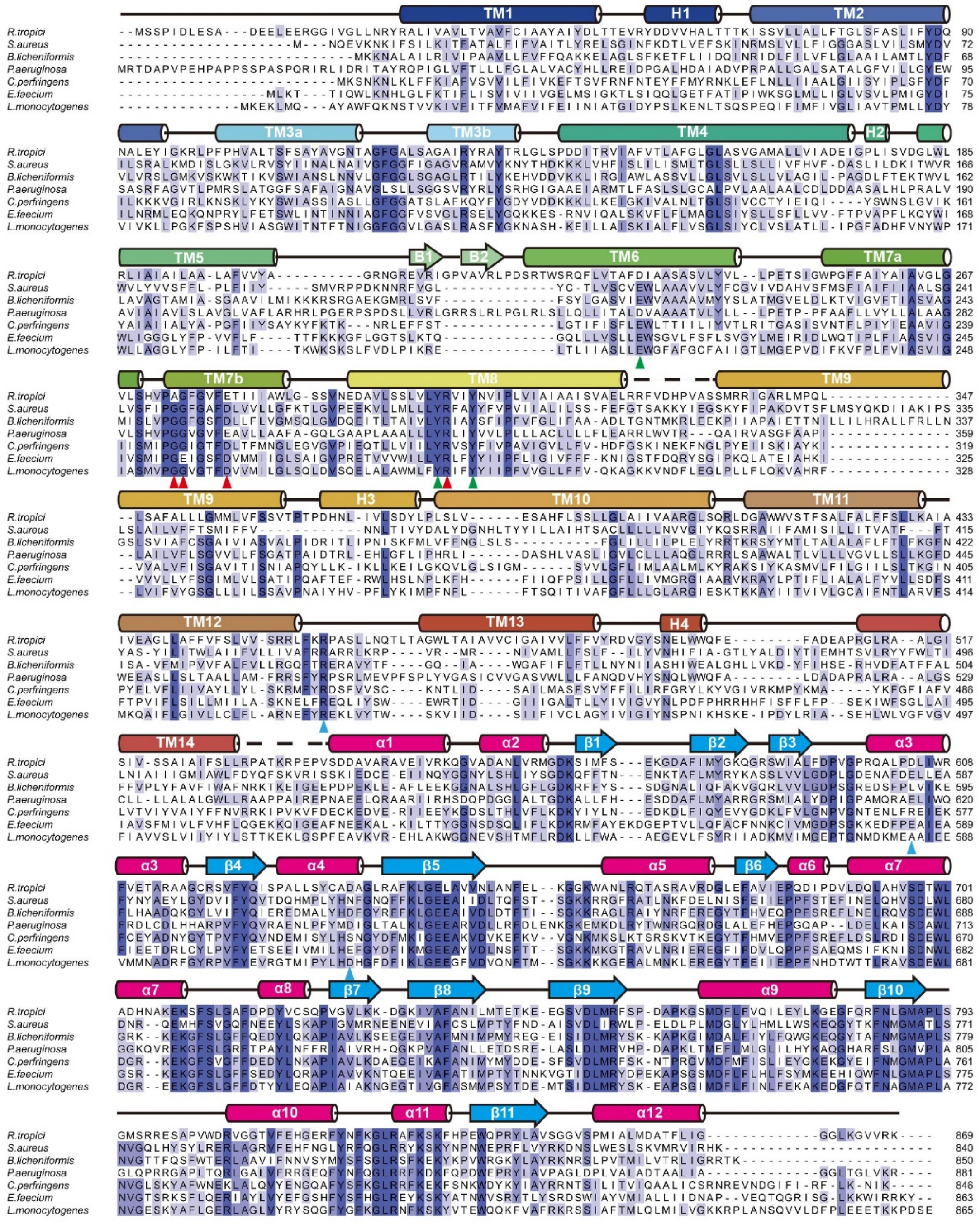


**Supplementary Figure 7. Alignment of the amino acid sequence of *Rt*MprF with the other homologs from different species.** The sequence alignment was accomplished by using Clustal Omega and presented by JalView. The α-helices and β-strands are indicated as cylinders and arrows respectively and are labeled above the sequence. The secondary elements of flippase domain are colored in rainbow colors. For the synthase domain, the α-helices and β-strands are colored in violet and cyan,

respectively. The red arrowheads indicate the conserved Glu/Asp-Arg pair serving as a barrier between Cavities C and P, while the green arrowheads label three conserved residues involved in binding the head group of LysPG. Conserved residues involved in the interactions between the flippase and synthase domains are indicated by blue arrowheads. *R. tropici*, *Rhizobium tropici*; *S. aureus, Staphylococcus aureus*; *B. licheniformis, Bacillus licheniformis*; *P. aeruginosa*, *Pseudomonas aeruginosa*; *C. perfringens*, *Clostridium perfringens (Cp*MprF2*)*; *E. faecium, Enterococcus faecium*; *L. monocytogenes, Listeria monocytogenes.*

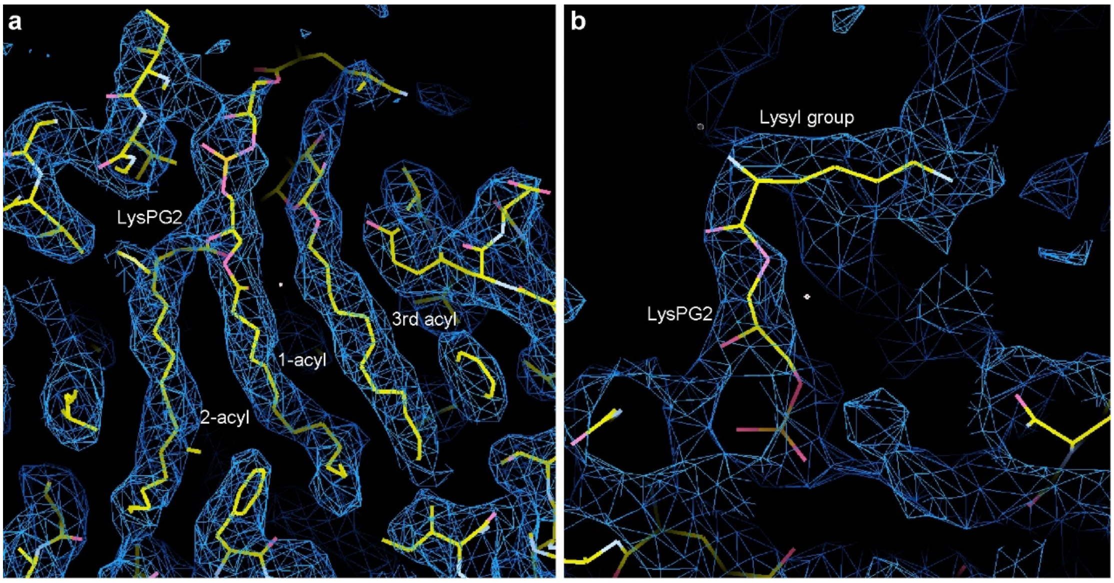


**Supplementary Figure 8. The cryo-EM densities for the lipid and detergent molecules in Cavity P of *Rt*MprF(GDN)-nanodisc. a**, The map contoured at 1.2×σ level shows three strong fatty acyl chains in Cavity P. **b**, The density feature potentially belonging to the lysyl group becomes visible when the map contour level is lowered at 0.8×σ.

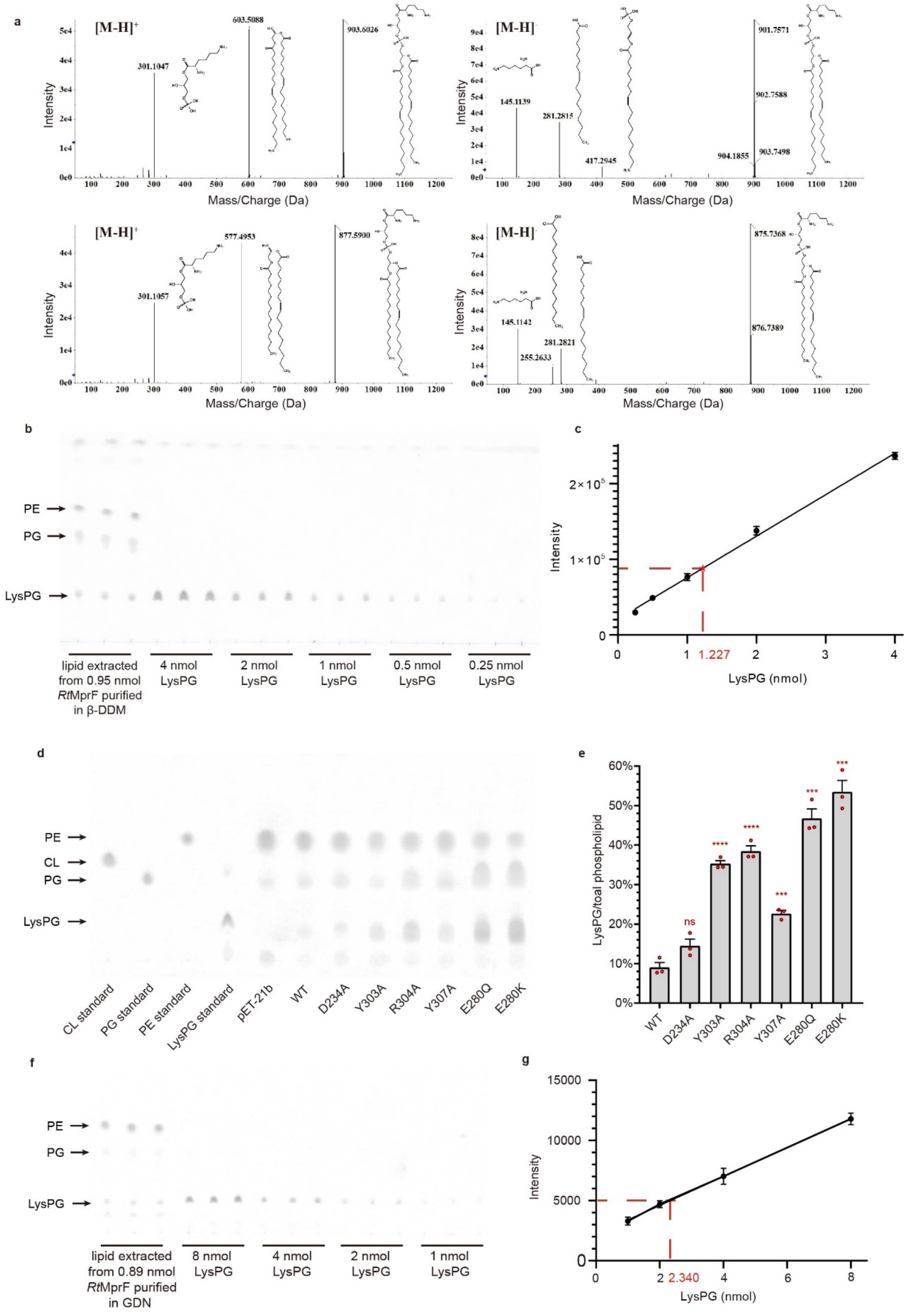


**Supplementary Figure 9. Identification and quantification of LysPG co-purified with *Rt*MprF protein. a,** Mass spectroscopy analysis of the lipids extracted from the purified *Rt*MprF protein sample. The mass spectrums in positive and negative modes

exhibit features characteristic of LysPG molecules. **b and c,** Quantification of the LysPG co-purified with *Rt*MprF(DDM) protein. The TLC plate was stained by iodine and the image shown in **b** was processed by Image J. The data extracted from the spots of the LysPG standard samples were used for linear regression of the standard curve ($Y = 54798 \times X + 20842$) as shown in **c**. By referring to the standard curve, the amount of LysPG extracted from 0.95 nmol *Rt*MprF protein purified in β-DDM is estimated to be 1.227 (corresponding to ~1.2 LysPG per *Rt*MprF monomer) as indicated by the red labels. The error bars in **c** denote the standard errors of the mean value (n = 3). **d**, TLC analysis of the overall total phospholipids extracted from *E. coli* cells expressing wild-type or various mutants of *Rt*MprF. The TLC plate was stained by iodine and the image was processed by ImageJ. By referring to the pure lipid standard samples of cardiolipin (CL), PG, PE and LysPG loaded on the left side, the three major spots in the lipid sample extracted from cells are identified as PE, PG and LysPG, respectively. **e**, The proportion of LysPG in total phospholipids extracted from *E. coli* cells expressing wild-type or various mutants of *Rt*MprF. The LysPG/total phospholipid ratio presented in the bar graphs (e) is calculated through dividing the LysPG spot intensity by the summed intensity of three major phospholipid spots (PE+PG+LysPG, approximating the total phospholipid quantity as the remaining ones like PS and CL are minor at low abundance) as shown on the TLC plate (d). The error bars in **e** indicate the standard errors of the mean values (n = 3). $p = 0.0583$ between WT and D234A, ns; $p = 0.00006$ between WT and Y303, ****; $p = 0.000087$ between WT and R304A, ****; $p = 0.000718$ between WT and Y307A, ***; $p = 0.000145$ between WT and E280Q, ***; $p = 0.000143$ between WT and E280K, *** (two-sided unpaired t-test between WT and various mutants). **f and g**, Quantification of the LysPG co-purified with *Rt*MprF(GDN) protein. The TLC plate was stained by iodine and the image shown in **f** was processed

by ImageJ. The data extracted from the spots of LysPG standard samples were used for linear regression of the standard curve ($Y = 1202 \times X + 2197$) as shown in **g**. By referring to the standard curve, the amount of LysPG extracted from 0.89 nmol *Rt*MprF protein purified in GDN is estimated to be 2.340 (corresponding to ~2.6 LysPG per *Rt*MprF monomer) as indicated by the red label. The error bars in **g** denote the standard errors of the mean values (n=3). Due to the difference in the pixel size setting of raw images (3264×2448 for b and 1360×1024 for f), the intensity values of the lipid spots presented in g are at different scale when compared to those shown in c. The intensity scale difference does not affect the final results of LysPG content measurement as the standard samples of LysPG are loaded on the same plate of the lipid samples under investigation. In case that the raw image is resized proportionally, the scale of lipid spot intensity values changes accordingly, whereas the measured LysPG amount remains very close to the one derived from raw data. For b, d and f, each experiment was repeated independently twice (b) or three times (d, f) with similar results.

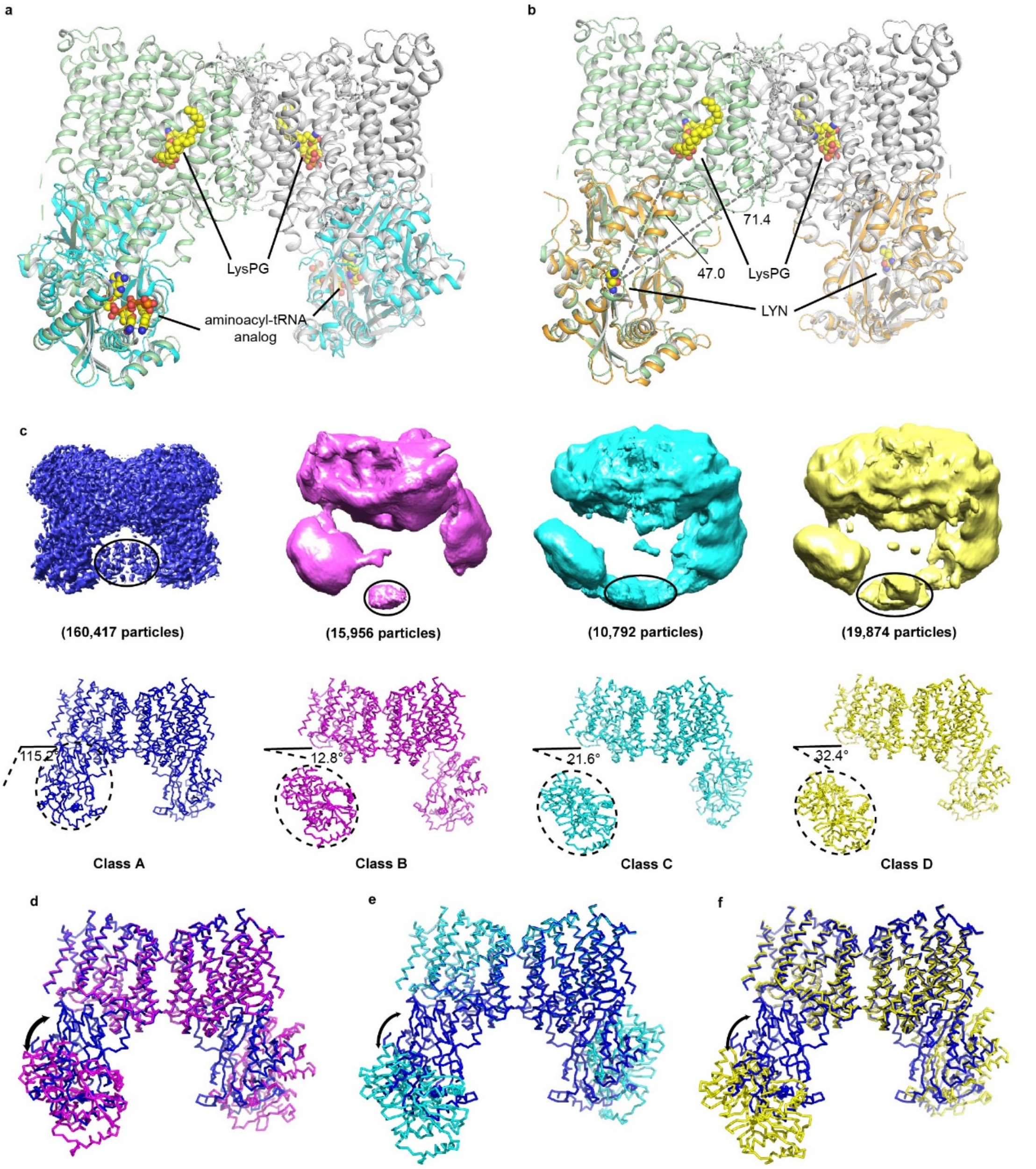


**Supplementary Figure 10. The relationship between the synthase and the flippase domains of *Rt*MprF. a**, Superposition of the structure of FemX-aminoacyl-tRNA complex [PDB code: 4II9 (https://www.rcsb.org/structure/4II9)] on one monomer of *Rt*MprF dimer. It is apparent that the active site in the synthase domain of *Rt*MprF is distant from the LysPG-binding site in the transmembrane domain. **b,** Superposition of the structure of *Bl*MprF-LYN complex [PDB code: 4V36 (https://www.rcsb.org/structure/4V36)] on *Rt*MprF dimer**.** The *Rt*MprF versus FemX and *Bl*MprF superposition leads to root mean square deviation of $\alpha$-carbon atoms at 3.1

and 2.2 Å respectively. **c**, The presence of multiple conformations of *Rt*MprF dimer. Three minor classes (Classes B-D) of asymmetrical *Rt*MprF dimer with conformations distinct from the major class of symmetrical dimer (Class A, used for solving the structure at 3.7 Å resolution) are shown. The cryo-EM densities are shown in the upper row, while the corresponding models are presented in the lower row. Color codes: blue, class A; magenta, class B; cyan, class C; yellow, class D. The regions covered by the solid elliptical rings are densities from the adjacent particles. In the structural models, the synthase domains exhibiting variable positions in respect to the transmembrane domain are labeled with dashed circles. The angles shown nearby are between the presumed membrane plane and the long axis of the synthase domain. **d-f,** Superposition of classes B (d), C (e) and D (f) with class A. The arrows indicate the predicted transition between the two different states.

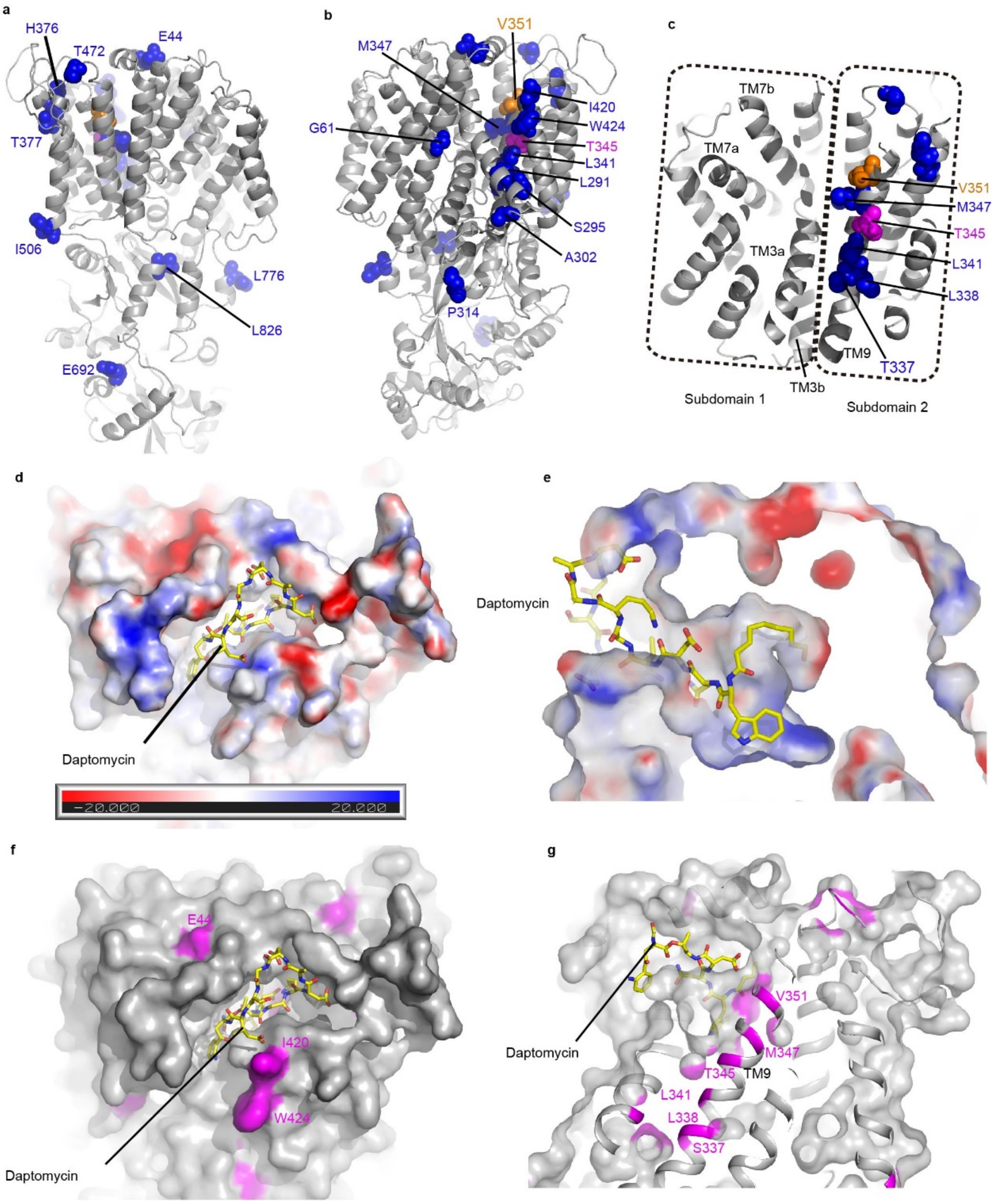


**Supplementary Figure 11. Structural model of *Sa*MprF and the locations of point mutation sites found in the DAP-R *S. aureus* strains. a and b,** Two different side views of *Sa*MprF model with the mutation sites highlighted in colored sphere models. Color codes: orange and magenta, two gain-of-function mutations causing DAP-R; blue, the other mutations found in the DAP-R strains. **c,** zoom-in-view of the mutation sites clustering on TM9 in Subdomain 2. **d** and **e**, Top view (d) and sectional side view (e) of the model of daptomycin molecule docked in Cavity E of *Sa*MprF. While

daptomycin is shown as stick models, *Sa*MprF is presented as electrostatic potential surface model. Unit, $kTe^{-1}$; color codes: red, electronegative; white, neutral; blue, electropositive. The affinity of daptomycin with *Sa*MprF is -10.8 kcal/mol (estimated by the Autodock program). **f** and **g**, Clustering of numerous DAP-R-related mutation sites around the putative daptomycin-binding cavity.

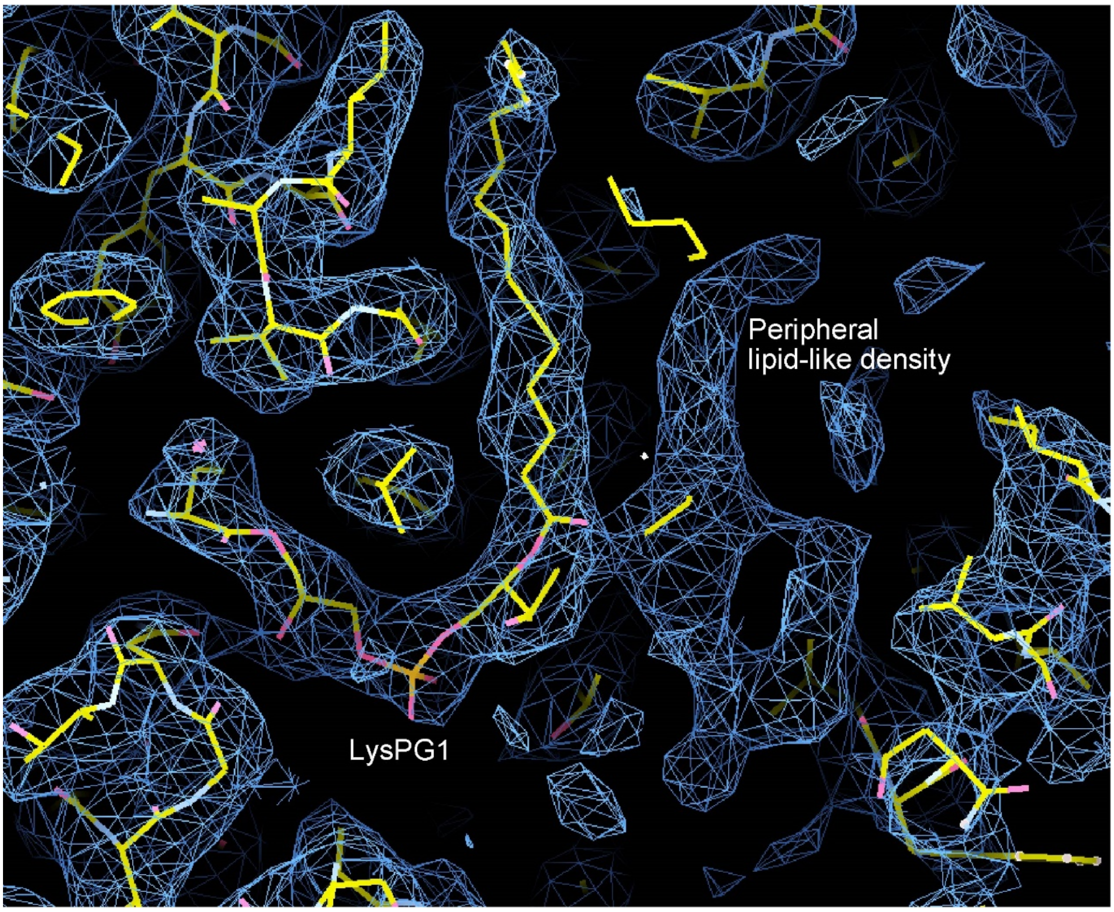


**Supplementary Figure 12. A peripheral lipid-like density nearby LysPG1 and at the cytoplasmic entrance of Cavity C.** The *Rt*MprF(GDN)-nanodisc map is contoured at 1.2 × σ level and superposed with the stick models.

**Supplementary Table 1. Cryo-EM data collection, single particle analysis and model building statistics of *Rt*MprF.**

| | ***Rt*MprF(DDM)-nanodisc** [EMDB-0992 (https://www.emdataresource.org/EMD-0992)] [PDB: 6LVF (https://www.rcsb.org/structure/6LVF)] | ***Rt*MprF(GDN)-nanodisc** [EMDB-30869 (https://www.emdataresource.org/EMD-30869)] [PDB: 7DUW (https://www.rcsb.org/structure/7DUW)] |
|---|---|---|
| **Data collection and processing** | | |
| Magnification | 130,000 | 130,000 |
| Voltage (kV) | 200 | 200 |
| Electron exposure ($e^-$/Å) | 50 | 50 |
| Defocus range (μm) | -1.5 to -2.0 | -1.5 to -2.0 |
| Pixel size (Å) | 1 | 1 |
| Symmetry imposed | *C*2 | *C*2 |
| Initial particle images (no.) | 887,196 | 1,232,621 |
| Final particle images (no.) | 160,417 | 144,479 |
| Map resolution (Å) | 3.70 | 2.96 |
| FSC threshold | 0.143 | 0.143 |
| Map resolution range (Å) | 3.2-6.0 | 2.2-3.8 |
| **Refinement** | | |
| initial model used (PDB code) | - | - |
| Model resolution (Å) | 3.74 | 3.05 |
| FSC threshold | 0.5 | 0.5 |
| Model resolution range (Å) | - | - |
| Map sharpening *B* factor (Å$^2$) | -170 | -62 |
| Model composition | | |
| Non-hydrogen atoms | 12130 | 13276 |
| Protein residues | 1586 | 1640 |
| Water | - | 2 |
| Ligands | 6 (1 LysPG, 2 PG /monomer) | 12 (2 LysPG, 2 PG, 1 GDN, 1 DDM/monomer) |
| *B* factors (Å$^2$) | | |
| Protein | 67.84 | 52.98 |
| Ligand | 41.95 | 41.02 |
| R.m.s. deviations | | |

| | | |
|---|---|---|
| Bond lengths (Å) | 0.010 | 0.004 |
| Bond angles (°) | 1.100 | 0.664 |
| Validation | | |
| MolProbity score | 1.83 | 1.93 |
| Clashscore | 5.89 | 14.13 |
| Poor rotamers (%) | 0.16 | 0.15 |
| Ramachandran plot | | |
| Favored (%) | 91.38 | 96.01 |
| Allowed (%) | 8.62 | 3.99 |
| Disallowed (%) | 0 | 0 |

## Supplementary Table 2. Strains, Plasmids and Primers

**_E. coli_ Strains used for clone screening and protein expression**

| *Strain* | *Usage* |
|---|---|
| DH5α | Clone screening |
| BL21 (DE3) | Expression of the synthase domain of *Rt*MprF |
| C41 (DE3) | Expression of the full length *Rt*MprF, *Rt*MprF D234A/Y303A/R304A/E280Q/E280K/D753A mutant, *RtPa*MprF |

**Plasmids used for protein expression**

| *Plasmid* | *Gene* | *Restriction site* |
|---|---|---|
| pET21b | the synthase domain of *Rt*MprF, Full length *Rt*MprF, *Rt*MprF D234A/Y303A/R304A/E280Q/E280K/D753A mutant, *RtPa*MprF | Nde I, Hind III |

**Primers used for plasmid construction**

| *Construct* | *Primer Type* | *Primer Sequence* |
|---|---|---|
| *Rt*MprF | Forward primer | 5’-GATATGACCATATGTCGAGCCCAATCGATC-3’ |
| | Reverse primer | 5'-ATGTTAAGCTTTTTCCTCACGACCCCCT- 3' |
| *Rt*MprF-Synthase | Forward primer | 5’-GGGAATTCCATATGGACGATGCGGTG-3’ |
| | Reverse primer | 5’-CCCAAGCTTTCCGCCCCCGAT-3’ |
| *RtPa*MprF | Forward primer | 5’-CCGGCAACGAAGCGGCCGGAGCCTGTCAGCGCGGAAGAGCTG-3’ |
| | Reverse primer | 5’-GTGGTGGTGCTCGAGTGCGGCCGCAAGCTTGCGTTTCACCAA-3’ |
| *Rt*MprF-D234A | Forward primer | 5’-CACCGCATTCGCGATCGCGGCCTCG-3’ |
| | Reverse primer | 5’-CGAGGCCGCGATCGCGAATGCGGTG-3’ |
| *Rt*MprF-Y303A | Forward primer | 5’-GTTCTCTGGTCCTCGCGCGCGTCATCTACAAC-3’ |
| | Reverse primer | 5’-GTTGTAGATGACGCGCGCGAGGACCAGAGAAC-3’ |
| *Rt*MprF-R304A | Forward primer | 5’-CTGGTCCTCTATGCGGTCATCTACAAC-3’ |
| | Reverse primer | 5’-GTTGTAGATGACCGCATAGAGGACCAG-3’ |
| *Rt*MprF-Y307A | Forward primer | 5’-CTATCGCGTCATCGCGAACGTCATTCC-3’ |
| | Reverse primer | 5’-GGAATGACGTTCGCGATGACGCGATAG-3’ |
| *Rt*MprF-E280Q | Forward primer | 5’-CTTCGGCGTGTTCCAGACGATCATC-3’ |
| | Reverse primer | 5’-GATGATCGTCTGGAACACGCCGAAG-3’ |
| *Rt*MprF-E280K | Forward primer | 5’-CTTCGGCGTGTTCAAAACGATCATCATC-3’ |
| | Reverse primer | 5’-GATGATGATCGTTTTGAACACGCCGAAG-3’ |
| *Rt*MprF-D753A | Forward primer | 5’-AGGGATCCGTCGCGCTGATGCGTTTTT-3’ |
| | Reverse primer | 5’-AAAAACGCATCAGCGCGACGGATCCCT-3’ |
