# Supplementary material for: Phospholipid translocation captured in a bifunctional membrane protein MprF
Source: Nat Commun. 2021 May 18;12:2927. doi: 10.1038/s41467-021-23248-z (PMC8131360; doi:10.1038/s41467-021-23248-z)
Supplement: Supplementary file 3 — Reporting Summary [file 41467_2021_23248_MOESM3_ESM.pdf]

nature research

Corresponding author(s): Zhenfeng Liu

Last updated by author(s): Apr 12, 2021

Reporting Summary

Nature Research wishes to improve the reproducibility of the work that we publish. This form provides structure for consistency and transparency in reporting. For further information on Nature Research policies, see our Editorial Policies and the Editorial Policy Checklist.

Statistics

For all statistical analyses, confirm that the following items are present in the figure legend, table legend, main text, or Methods section.

n/a	Confirmed	
☐	☒	The exact sample size (*n*) for each experimental group/condition, given as a discrete number and unit of measurement
☐	☒	A statement on whether measurements were taken from distinct samples or whether the same sample was measured repeatedly
☐	☒	The statistical test(s) used AND whether they are one- or two-sided<br>*Only common tests should be described solely by name; describe more complex techniques in the Methods section.*
☒	☐	A description of all covariates tested
☒	☐	A description of any assumptions or corrections, such as tests of normality and adjustment for multiple comparisons
☐	☒	A full description of the statistical parameters including central tendency (e.g. means) or other basic estimates (e.g. regression coefficient) AND variation (e.g. standard deviation) or associated estimates of uncertainty (e.g. confidence intervals)
☐	☒	For null hypothesis testing, the test statistic (e.g. *F*, *t*, *r*) with confidence intervals, effect sizes, degrees of freedom and *P* value noted<br>*Give P values as exact values whenever suitable.*
☒	☐	For Bayesian analysis, information on the choice of priors and Markov chain Monte Carlo settings
☒	☐	For hierarchical and complex designs, identification of the appropriate level for tests and full reporting of outcomes
☒	☐	Estimates of effect sizes (e.g. Cohen's *d*, Pearson's *r*), indicating how they were calculated

Our web collection on statistics for biologists contains articles on many of the points above.

Software and code

Policy information about availability of computer code

Data collection	SerialEM 3.6.2 was used for collecting cryo-EM images. The X-ray diffraction datasets were collected by using software UGUI 2 in Photon Factory beamline BL-1A.
Data analysis	HKL2000, CCP4 6.0.2, Phenix 1.11.1, Coot 0.8.9.2, Image J 1.8.0, GraphPad Prism 8.0.2, PyMOL 2.3.2, CTFFIND 4.1.15, cryoSPARC 2.9, RELION 3.0, UCSF Chimera 1.9, Resmap 1.1.4, Image J 1.8.0, GraphPad Prism 8.0.2, TMHMM 2.0, PSIPRED 4.0

For manuscripts utilizing custom algorithms or software that are central to the research but not yet described in published literature, software must be made available to editors and reviewers. We strongly encourage code deposition in a community repository (e.g. GitHub). See the Nature Research guidelines for submitting code & software for further information.

Data

Policy information about availability of data

All manuscripts must include a data availability statement. This statement should provide the following information, where applicable:

- Accession codes, unique identifiers, or web links for publicly available datasets
- A list of figures that have associated raw data
- A description of any restrictions on data availability

The structural models have been deposited in the Protein Data Bank under accession codes of 6LVF (https://www.rcsb.org/structure/6LVF) and 7DUW (https://www.rcsb.org/structure/7DUW) for RtMprF(DDM)-nanodisc and RtMprF(GDN)-nanodisc, and 6LV0 (https://www.rcsb.org/structure/6LV0) for the synthase domain of RtMprF. The cryo-EM density maps of RtMprF(DDM)-nanodisc and RtMprF(GDN)-nanodisc have been deposited in the Electron Microscopy Data Bank under accession code EMD-0992 (https://www.emdataresource.org/EMD-0992) and EMD-30869 (https://www.emdataresource.org/EMD-30869). The other PDB files used for analysis and comparison in this work were downloaded from the RCSB PDB website (http://www.rcsb.org/), including the structures of daptomycin [accession code: 1T5M (https://www.rcsb.org/structure/1T5M)], FemX-aminoacyl-tRNA complex [accession code: 4II9 (https://www.rcsb.org/structure/4II9)],

BlMprF-LYN complex structure [accession code: 4V36 (https://www.rcsb.org/structure/4V36)], TMEM16F [accession code: 6QP6 (https://www.rcsb.org/structure/6QP6)], MurJ [accession code: 6NC9 (https://www.rcsb.org/structure/6NC9)], MsbA [accession code: 5TV4 (https://www.rcsb.org/structure/5TV4)] and Drs2p-Cdc50p [accession code: 6ROJ (https://www.rcsb.org/structure/6ROJ)]. Figure 1e, Figure 3e & f, Figure 4b, Figure 4c, Figure 4d & e, Figure 4f & g, Figure 4h & l, Figure 4j & k, Supplementary Fig. 1a, Supplementary Fig. 2a, Supplementary Fig. 9b & c, Supplementary Fig. 9d & e and Supplementary Fig. 9f & g have associated raw data. The source data of western blots, SDS-PAGE and TLC are provided in the Source Data file with this paper. All data are available in the online version of the paper or upon request.

Field-specific reporting

Please select the one below that is the best fit for your research. If you are not sure, read the appropriate sections before making your selection.

- [x] Life sciences
- [ ] Behavioural & social sciences
- [ ] Ecological, evolutionary & environmental sciences

For a reference copy of the document with all sections, see nature.com/documents/nr-reporting-summary-flat.pdf

Life sciences study design

All studies must disclose on these points even when the disclosure is negative.

Sample size	No statistical methods were used to predetermine sample size. The single particle cryo-EM analysis averaged 160,417 and 144,479 particles for the reported RtMprF(DDM)-nanodisc and RtMprF(GDN)-nanodisc structures, respectively. The sample size was chosen according to the previous theoretical study indicating that a minimal number of 12,000 single particles is required for determining a protein structure at 3 Å resolution through the single-particle cryo-EM method (References: Henderson, R. The potential and limitations of neutrons, electrons and X-rays for atomic resolution microscopy of unstained biological molecules. Q. Rev. Biophys. 28: 2, 1995; Vinothkumar, K. R. and Henderson, R. Single particle electron cryomicroscopy: trends, issues and future perspective. Q. Rev. Biophys. 49: e13, 2016).
Data exclusions	The initial cryoEM raw images were screened manually and the micrographs with low-resolution Thon ring profiles and severe ice contamination were excluded. During 2D and 3D classification of the particle images, the classes with poorly defined internal features were excluded.
Replication	For quantification of lipid through TLC, the experiments were performed with three repeats for each sample. Each experiment was replicated at least twice on separate occasions. All attempts at replication were successful.
Randomization	Randomization is irrelevant for our study because no grouping was needed. For single-particle cryo-EM analysis, particles are divided into two random sets for map reconstruction and estimation of the resolution via the Fourier Shell Correlation (FSC) method.
Blinding	Blinding is irrelevant to our study because no allocation into experimental groups was needed.

Reporting for specific materials, systems and methods

We require information from authors about some types of materials, experimental systems and methods used in many studies. Here, indicate whether each material, system or method listed is relevant to your study. If you are not sure if a list item applies to your research, read the appropriate section before selecting a response.

Materials & experimental systems

n/a	Involved in the study	
☐	☒	Antibodies
☒	☐	Eukaryotic cell lines
☒	☐	Palaeontology and archaeology
☒	☐	Animals and other organisms
☒	☐	Human research participants
☒	☐	Clinical data
☒	☐	Dual use research of concern

Methods

n/a	Involved in the study	
☒	☐	ChIP-seq
☒	☐	Flow cytometry
☒	☐	MRI-based neuroimaging

Antibodies

Antibodies used	Anti-His Mouse monoclonal antibody; TransGen Biotech (HT501)<br>Goat anti-Mouse IgG (H+L)-HRP; Beijing Lablead Biotech (S0100)
Validation	Anti-His Mouse monoclonal antibody (TransGen Biotech. Cat. # HT501)<br>Validation statement from manufacturer's website:<br>Anti-His Mouse Monoclonal Antibody is a purified monoclonal antibody that detects recombinant proteins containing the 6×His (HHHHHH) epitope tag.<br>Goat anti-Mouse IgG (H+L)-HRP (Beijing Lablead Biotech. Cat. # S0100)<br>Validation statement from manufacturer's website:

The secondary antibody is improved based on the original production and have a strong reaction with the IgG heavy and light chains. Meanwhile, the secondary antibody have low reaction with human IgG through adsorption human serum protein. Compared With ordinary secondary antibody, our antibody with high sensitivity and specificity and so on. And application for Western blot, enzyme-linked immunosorbent assay (Elisa) and other experiments.
